# New Publications

**Published:** 1897-04

**Authors:** 


### NEW PUBLICATIONS.

The translation of Professor Georg Müller's *Diseases of the Dog* is rapidly nearing completion. Dr. Alexander Glass, the translator, is sparing no effort to have the book complete in every detail and brought down to the latest researches and investigations of the diseases of canines. The illustrations, numbering nearly one hundred, will add greatly to the value of the book. The work is being printed by the well-known medical printing house of William J. Dornan, printer of the JOURNAL, an assurance of the best workmanship.

The *Agricultural Journal*, of the Cape of Good Hope, comes out in its January issue in new form—a complete new dress, more attractive, more readable, and is a very acceptable change over the former issues of this periodical.

